# Cocaethylene: When Cocaine and Alcohol Are Taken Together

**DOI:** 10.7759/cureus.22498

**Published:** 2022-02-22

**Authors:** Joseph Pergolizzi, Frank Breve, Peter Magnusson, Jo Ann K LeQuang, Giustino Varrassi

**Affiliations:** 1 Cardiology, Native Cardio Inc., Naples, USA; 2 Pharmacy, Temple University, Philadelphia, USA; 3 Department of Cardiology, Center of Research and Development Region Gävleborg/Uppsala University, Gävle, SWE; 4 Department of Medicine, Cardiology Research Unit, Karolinska Institutet, Stockholm, SWE; 5 Pain Management, NEMA Research, Inc., Naples, USA; 6 Pain Management, Paolo Procacci Foundation, Rome, ITA

**Keywords:** polysubstance use disorder, ethanol, alcohol, cocaine, cocaethylene

## Abstract

Cocaine is taken frequently together with ethanol and this combination produces a psychoactive metabolite called cocaethylene which has similar properties to the parent drug and may be more cardiotoxic. Cocaethylene has a longer half-life than cocaine, so that people who combine cocaine and ethanol may experience a longer-lasting, as well as more intense, psychoactive effect. Cocaethylene is the only known instance where a new psychoactive substance is formed entirely within the body. Although known to science for decades, cocaethylene has not been extensively studied and even its metabolic pathways are not entirely elucidated. Like its parent drug, cocaethylene blocks the reuptake of dopamine and increases post-synaptic neuronal activity; the parent drug may also block reuptake of serotonin as well. Cocaethylene has been studied in animal models in terms of its pharmacology and its potential neurological effects. Since the combination of cocaine and alcohol is commonly used, it is important for clinicians to be aware of cocaethylene, its role in prolonging or intensifying cocaine intoxication, and how it may exacerbate cocaine-induced cardiovascular disorders. Most cardiac-related risk assessment tools do not ask about cocaine use, which can prevent clinicians from making optimal therapeutic choices. Greater awareness of cocaethylene is needed for clinicians, and those who use cocaine should also be aware of the potential for polysubstance use of cocaine and ethanol to produce a potentially potent and long-lasting psychoactive metabolite.

## Introduction and background

Cocaine is a sympathomimetic that affects a variety of receptors in the body, releasing specific catecholamine and blocking their reuptake at certain sites. In the short term, cocaine acts as a vasoconstrictor and subjects who use cocaine present with dilated pupils, elevated body temperatures, rapid heart rates, and high blood pressure. At higher doses, cocaine may induce behavioral changes including paranoia, aggression, and violence; cocaine has potentially life-threatening cardiotoxic effects [1]. When cocaine and ethanol are used together, a psychoactive metabolite is produced with similar pharmacological and psychoactive properties as cocaine [2]. This metabolite, cocaethylene, is considered more toxic to the cardiovascular and hepatic systems than cocaine, the parent drug, and it has a longer plasma elimination half-life (about 2 hours) than cocaine (about 1 hour) [3]. There are other metabolites produced as well but they go beyond the scope of this review. The serum concentration of cocaethylene is not readily predictable because it is based on the timing of the use of ethanol with cocaine and the quantities used [3]. While cocaethylene is often encountered in clinical work, it has not been the subject of extensive investigation and there may be a lack of clinical understanding of this metabolite and its role in overdose toxicity, cocaine-induced heart disease, and drugged driving [3].

Polysubstance use disorder is prevalent among recreational drug users, including cocaine users, and ethanol is frequently combined with cocaine [4]. Cocaine is a stimulant that can produce feelings of euphoria, but as it wears off anxiety may arise; alcohol is sometimes used with cocaine to enhance the effects of cocaine, prolong the cocaine high, or to soften the sometimes abrupt “bumps” in cocaine use [5,6]. Some people using cocaine may take alcohol for no other reason than it is available at the time.

In a study of 2,016 intoxicated drivers who submitted to drug and alcohol testing, 6.0% (n=131) were polysubstance users and, of that group, 5.6% were using cocaine and alcohol concurrently [7]. A prospective study of 417 trauma patients ≥13 years found that 8.9% had cocaethylene metabolites in their system, indicating the concurrent use of cocaine plus ethanol. In this study of people seeking medical care for trauma, the presence of cocaethylene significantly increased the probability of intensive care unit (ICU) admission (odds ratio 5.9) [8]. In another single-center study of 15 male trauma patients, 13/15 had detectable cocaethylene in their system upon admission [9].

While cocaethylene is detectable in many drug assays and is well known to forensic professionals, clinicians may be less aware of cocaethylene and its possible effects on the clinical outcomes of cocaine-using patients. The goal of our narrative review is to present a clinically relevant overview of the cocaethylene metabolite. The PubMed database of the National Institute of Medicine was searched in August 2021 for “cocaethylene” with 473 results. When using PubMed, we also used the “similar articles” feature, when available, to find related articles. When the search was narrowed to the past five years, there were 63 results. The Embase database was searched and presented 542 results. The Web of Science database was searched with two results. Although the Cochrane Library is included in PubMed, it was searched independently, and there were 17 meta-analyses. This search formed the basis of the literature used in this report. Emphasis was placed on newer (<5 years) research but some important research about cocaethylene was conducted 20 or more years ago.

## Review

Cocaine is metabolized by carboxylesterase enzymes, hydrolyzing cocaine into two major metabolites: benzoylecgonine and ecgonine methyl ester [10]. When cocaine is taken in the presence of ethanol, the metabolic pathway of cocaine changes. Instead of hydrolysis with water, some of the cocaine undergoes transesterification with ethanol and produces cocaethylene [11]. Note that cocaethylene is the ethyl ester while cocaine is the methyl ester of benzoylecgonine. This is the only known instance where a new psychoactive substance is formed entirely within the body [2]. The combination of cocaine plus ethanol produces not just cocaethylene, but it also diverts the metabolism of cocaine into the inactive benzoylecgonine metabolite to the active cocaethylene metabolite [12].

In many ways, cocaethylene produces effects similar to those of cocaine. In a randomized, double-blind study of eight subjects, patients were administered intranasal cocaine or intranasal cocaethylene; subjects could not differentiate between equimolar doses of these two agents, both of which produced euphoric effects [13]. However, there are important pharmacodynamic differences. Compared to cocaine, cocaethylene had slower clearance, larger volume of distribution, and longer elimination half-life [10].

**Figure 1:**
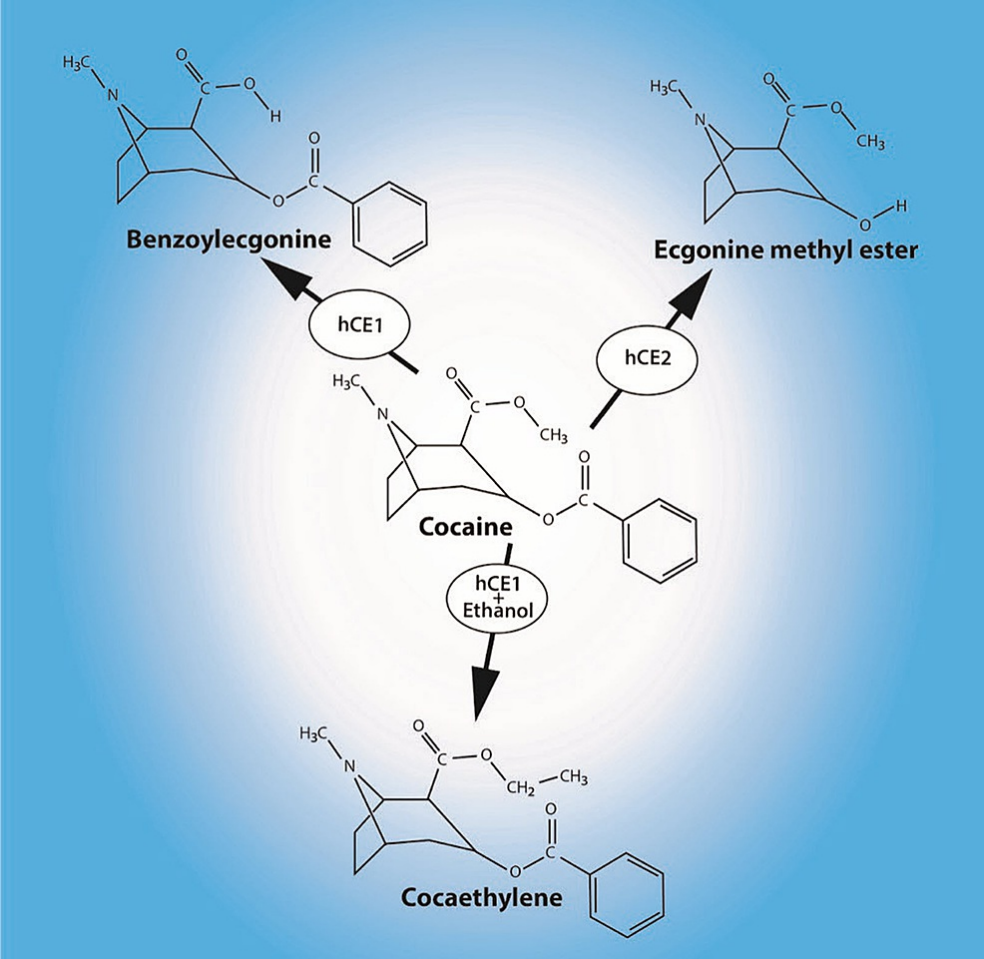
Putative metabolic pathways for cocaine and ethanol using the carboxylesterase 1 and 2 enzymes (hCE1 and hCE2).

The two main carboxylesterase enzymes in humans are carboxylesterase 1 (hCE1) and 2 (hCE2) mostly localized in the liver. These two isoforms are different gene products [14]. The hCE1 enzyme metabolizes certain endogenous esters, some pharmacological agents, and insecticides, while hCE2 metabolizes anticancer agents, such as capecitabine [15]. The hCE1 and hCE2 enzymes are high-capacity, low-affinity enzymes with the ability to hydrolyze structurally dissimilar esters to transform lipophilic esters into more water-soluble alcohol and acyl substituents [16]. Although it was long believed that cocaine was metabolized into benzoylecgonine via hCE1 and into ecgonine methyl ester via hCE2, this has come under question [17]. The hCE1 enzyme appears to be a broad-spectrum bio-scavenger and is known to play a role in the metabolism of biotoxins such as sarin [18]. See Figure 1.

The cocaine and cocaethylene molecules are structurally and chemically similar. It is not clear how cocaethylene is further metabolized, but a study in dogs found that cocaethylene clearance decreases when both cocaine and cocaethylene are present, suggesting that cocaethylene has a higher affinity for the active enzyme sites of cocaine [19]. See Table 1. However, when cocaine alone or cocaethylene alone was taken with ethanol, clearances of both were similarly decreased by approximately 20%. Over the course of time, benzoylecgonine achieves similar concentrations in the serum following the use of cocaine alone or cocaethylene alone, suggesting that benzoylecgonine is the major metabolite of cocaethylene as well as cocaine [19].

**Table 1:** From a canine study comparing cocaine alone and cocaethylene alone, the pharmacokinetic dispositions were similar for both agents [19].

	Cocaine alone	Cocaethylene alone
Clearance	0.91 ± 0.22 L/min	0.79 ± 0.16 L/min
Volume distribution	2.6 ± 0.82 L/kg	2.7 ± 0.47 L/kg

In a study of 10 healthy volunteers administered a continuous intravenous (IV) infusion of cocaine along with a single oral dose of ethanol 1 g/kg or an IV placebo and ethanol or IV cocaine and an ethanol placebo in a crossover design, it was found that 17% ± 6% of the cocaine was converted to cocaethylene in subjects who took cocaine with ethanol. Ethanol decreased the amount of benzoylecgonine excreted in the urine by 48%. In this study, subjects reported that the combination of cocaine plus ethanol was more intoxicating and pleasurable than either agent alone [20]. 

Dopamine is a reinforcing substance that plays a key role in the effects of many drugs of abuse, including cocaine and alcohol [21]. Independent of the route of administration, the initial effect of cocaine on the body is a rapid build-up of dopamine [22]. Dopamine originates in the dopaminergic cells of the brain and circulates throughout the body [22]. Circulating dopamine molecules can attach to receptor cells and, in that way, stimulate specific responses. An appropriate number of dopamine molecules are needed at any given time to activate these receptors appropriately, and this dopamine balance is systematically regulated by the brain. Cocaine actively interferes with the brain’s efforts to achieve and maintain optimal dopamine levels by occupying the dopamine transporters, the proteins by which the brain’s dopamine-producing cells use to retrieve excess dopamine molecules [23]. By allowing these dopamine molecules to accumulate, the dopamine receptors become excessively stimulated [22]. In this context, it is important to remember that the neural circuits affected by cocaine are considered fundamental biological pathways essential for survival [22].

Central to the psychoactive effects of cocaine is the nucleus accumbens (NA) region of the brain, a major part of the ventral striatum that helps to mediate emotions, motivation, reward, and pleasure. As an adaptive response aimed at keeping humans productive and active in functions that promote their survival, dopamine’s effect on the NA is to produce feelings of well-being, satisfaction, and/or pleasure in particular contexts, such as during sexual activity. In another example, a person with great thirst who is given water will experience a rush of dopamine to the NA. Cocaine can cause dopamine build-up and dopamine activity in the NA that exceeds the levels of dopamine that might occur naturally [24]. An interesting phenomenon is the fact that memory centers of the brain, the hippocampus and amygdala, work together with dopamine release from the NA so that the brain is vividly imprinted with cues to help identify the source of the pleasure. For example, a thirsty person given water will remember the event, the source of the water, and the experience of drinking. Repeated cocaine use causes associations to form in the brain that try to link cocaine-induced delivery of dopamine to the external circumstances. This is thought to play an important role in cocaine addiction [22].

Cocaethylene, like its parent drug cocaine, blocks the reuptake of dopamine and increases post-synaptic neuronal activity and reinforces the stimulating effects of dopamine [3,11]. This neuronal stimulation contributes to the powerful psychoactive effects of both cocaine and cocaethylene, producing feelings of energy, focus, and excitement. In terms of this central stimulatory effect, cocaine and cocaethylene appear to be equipotent [13], but cocaethylene’s longer half-life makes its effects more persistent [25]. This ability to prolong the cocaine high may help explain why many cocaine users drink alcohol while using cocaine, even if they are unaware of extending cocaine’s psychoactive effects by consuming ethanol [26].

Cocaethylene seems to be far more selective to dopaminergic sites than cocaine [11], since the latter appears to block serotonin reuptake as well as dopamine reuptake [2]. Both cocaine and cocaethylene increase the post-synaptic neuronal activity in an equipotent fashion although the effects of cocaethylene are more enduring [3].

The cardiovascular effects of cocaethylene

Cocaethylene increases heart rate and blood pressure more than cocaine [27]. In fact, cocaethylene is thought to be over 10 times more cardiotoxic than cocaine [28]. Murine studies have confirmed that cocaethylene is both more potent and longer lasting than the parent drug [29]; a canine study found that cocaethylene was also more cardiotoxic [30]. In a study of 23 dogs, animals were randomized to receive one of the following: IV bolus doses of cocaine 7.5 mg/kg plus ethanol 1 g/kg; cocaine boluses only; ethanol boluses only; or placebo. Two of the eight dogs in the cocaine plus ethanol group suffered cardiovascular collapse during the study. Cocaine plus ethanol had greater cardiotoxicity than either cocaine or ethanol alone [28]. However, there are few studies specifically addressing cocaethylene, and the acute cardiotoxic effects of cocaethylene in humans are not known [31].

Because cocaethylene is a more potent sodium channel blocker than cocaine, it seems plausible that it would have more severe adverse effects on the cardiovascular system [25]. Sodium channel blockers can reduce the speed of action potential transmission in the heart, reducing conduction velocity [25]. In a study in which six healthy volunteers insufflated 2 mg/kg cocaine and then without delay consumed 1 g/kg ethanol, it was found that after consuming both drugs, all subjects had heart rates significantly elevated over baseline [32]. However, in a study of six healthy males who received an IV bolus injection of either cocaine (0.25 mg/kg) or cocaethylene (0.25 mg/kg), the drug-induced elevation of heart rate and blood pressure in the first 2 hours after injection were greater with cocaine alone than with cocaethylene alone [33].

Cocaethylene and trauma

Cocaine intoxication increases the risk and worsens outcomes for trauma patients because cocaine has adverse hemodynamic and systemic effects which can exacerbate morbidity and even increase mortality in trauma patients [34,35]. Pre-injury substance use in general is associated with higher complications following trauma [36]. The exact role cocaethylene plays remains unknown. In a study of 453 trauma patients (83.7% male) presenting for emergency medical attention at a single center, 15.9% tested positive for cocaethylene, 19.8% for cocaine alone, and 11% for crack alone. Those patients who took cocaine (with or without alcohol) had more severe traumas than those who had used crack [35]. The traumas were of various natures. The study did not analyze the specific outcomes for the cocaethylene patients. 

Neurological effects of cocaethylene

Although it had once been thought that neurons could not replicate in adults, it has been discovered that certain areas of the brain, that is, the dentate gyrus of the hippocampal formation and the subventricular zone, are capable of generating new neurons in adult humans [37]. The adult brain contains a small population of neural stem cells that help to repair and maintain cerebral tissue [38]. Adult mammals experience neurogenesis to a limited extent over the course of their lifetime [39]. This may be adversely affected by cocaethylene. In a study of mice, the long-term exposure of animals to ethanol and cocaine induced pathological changes in the brain and neurodegeneration [38].

Serum concentrations of cocaethylene

The serum concentrations of cocaethylene depend on both the amount and timing of the two agents (cocaine and ethanol) consumed. People who use cocaine without any ethanol would have no measurable amount of cocaethylene in their system, but the ingestion of even small amounts of ethanol may result in production of cocaethylene [3]. Likewise, people who consume ethanol but take no cocaine or very little cocaine would not produce cocaethylene [40]. The greatest cocaethylene production would theoretically occur in a person who has a relatively high blood-alcohol level at the point in which they used cocaine [40]. In real-world clinical practice, it can be very difficult to predict cocaethylene concentrations in the blood, even when the exact amounts and timing of alcohol and cocaine use are known. The longer half-life of cocaethylene means that its measurable presence in the blood indicates that the person had used cocaine, even if cocaine is no longer detectable [3].

Cocaethylene epidemiology

Drug and alcohol use disorders occur in all races, ethnicities, social-economic groups, sexes, and in young and old alike. While the use of cocaine and alcohol at the same time is a common form of polysubstance abuse, it along with other patterns of polysubstance use have not been well studied [41]. There are many reasons alcohol might be consumed with cocaine: to enhance or prolong the psychoactive effects of cocaine, to calm down after a cocaine binge, to soothe the “bumps” as cocaine inebriation wears off, to help induce sleep after a sustained period of activity, or even opportunistically because alcohol was available [42].

Polysubstance drugs users may use opioids as well; in fact, cocaine and opioid is a popular drug combination. A study of polysubstance users with heroin addiction participating in methadone maintenance programs identified 53 patients who were stabilized in the program and matched to controls who dropped out of the program or were noncompliant. The cessation of use of illicit opioids correlated to decreases in cocaine and alcohol use in these subjects [43]. In a study of 66 adults in a methadone maintenance program who reported cocaine use, about 60% of subjects said they often took alcohol to help ease the discomfort or unpleasant transitions involved in the use of cocaine or crack [42]. Among polysubstance users, the use of alcohol, opioids, and cocaine is particularly prevalent.

Clinical considerations

While many cocaine users take ethanol together with cocaine, few understand that it produces a new potent metabolite in the body that has a longer half-life and potentially more dangerous cardiotoxic effects. Cocaine users may feel that alcohol prolongs or enhances their “high” without knowing exactly why. In fact, greater education is needed for patients, their families, and illicit drug users. Patients should be informed about the risks of using cocaine and ethanol at the same time. The longer half-life of cocaethylene may allow some to overdose on cocaine, not realizing that the cocaethylene metabolite was still active.

Cocaine intoxication is frequently seen in emergency departments, but clinicians may treat cocaine-induced arrhythmias, myocardial infarction, or other adverse events and not ask about the concurrent use of cocaine and ethanol. A patient who admits to using cocaine but fails to mention alcohol consumption at the same time may be experiencing cocaine-like psychoactive effects long after the clinician would assume they had dissipated. Clinicians treating cocaine toxicity may find that the half-life of cocaine is approximately doubled when ethanol is used at the same time. Even if a patient is forthcoming about the quantity and timing of taking cocaine, the effects of the drug may be significantly prolonged when alcohol is involved. However, the concentration of cocaethylene produced by an individual using both cocaine and ethanol is highly dependent on the amount and sequence of these precursor drugs, that is to say, cocaethylene concentration cannot be readily predicted [3]. Clinicians treating patients with acute or chronic cocaine exposure should ask about alcohol consumption to get a more realistic assessment of their risk.

While the parent drug, cocaine, is associated with an elevated risk for cardiovascular disease, myocardial infarction, and arrhythmias [44,45], most risk assessment metrics for heart disease do not ask about cocaine use. In fact, except for the use of alcohol and tobacco, some potentially relevant substances are not mentioned in cardiovascular screening [46]. Further study is needed as to the nature of cocaethylene, its metabolic fate in the body, and its acute and chronic effects.

Route of administration may play a role. The oral route resulted in significantly greater cocaethylene formation than smoking (34% ± 20% versus 18% ± 11%, respectively) and showed a trend toward significance for greater formation of cocaethylene compared to even intravenous administration. Such information, when available from the patient or others, may be helpful for the emergency team [47].

## Conclusions

While the pharmacology of cocaethylene has been known for decades, its role in cocaine intoxication, cocaine-induced heart disease, and polysubstance use disorder has not been well studied. Clinicians may be unaware that the concurrent consumption of ethanol and cocaine may result in prolonged cocaine-like effects and may be more cardiotoxic than if the drugs are not taken at the same time. Greater awareness is needed among clinicians and patients; although it is always possible to predict serum concentrations of cocaethylene based on the amount and timing of their use, clinicians may be prudent to ask patients with cocaine-related adverse effects or toxicity about their alcohol use patterns. Greater study is needed about the effects and metabolic pathways of cocaethylene in the body.
